# Eosinophilic Colitis in an Adult Ethiopian Patient

**DOI:** 10.1155/crgm/1279031

**Published:** 2026-01-30

**Authors:** Kaleb Assefa Berhane, Ahmed Adem, Abate Bane Shewaye, Nathan Tolemariam Jibat, Lela Alemayehu Gebeyehu, Fadil Nuredin Abrar

**Affiliations:** ^1^ Department of Internal Medicine, Adera Medical and Surgical Center, Addis Ababa, Ethiopia; ^2^ Department of Internal Medicine, College of Health Sciences, Addis Ababa University, Addis Ababa, Ethiopia, aau.edu.et; ^3^ School of Medicine, St. Paul’s Hospital Millennium Medical College, Addis Ababa, Ethiopia, sphmmc.edu.et; ^4^ Department of Pathology, College of Health Sciences, Addis Ababa University, Addis Ababa, Ethiopia, aau.edu.et; ^5^ Department of Pathology, ONCO Pathology Diagnostic Center, Addis Ababa, Ethiopia

## Abstract

Eosinophilic colitis (EC) is a rare subtype of eosinophilic gastrointestinal disorders, marked by dense eosinophilic infiltration of the colon in the absence of secondary causes. We report the case of a 48‐year‐old Ethiopian man with a history of atopic disease who presented with chronic nonbloody diarrhea, intermittent abdominal pain, and fatigue. Diagnostic colonoscopy with biopsy confirmed EC, supported by peripheral eosinophilia and histological findings. The patient was treated with oral prednisolone, leading to complete symptom resolution.

## 1. Introduction

Eosinophilic gastrointestinal diseases (EGIDs) are rare, clinicopathologically defined disorders characterized by prominent eosinophilic infiltration of the gastrointestinal (GI) tract. These conditions are classified based on the primary site of involvement and include eosinophilic esophagitis, eosinophilic gastritis, eosinophilic duodenitis, eosinophilic gastroenteritis, and eosinophilic colitis (EC). Among these, EC represents the least common and least well‐characterized subtype, with an estimated prevalence of 1.6–2.1 cases per 100,000 individuals [1–3]. It predominantly affects females in the adult population and displays a bimodal age distribution, most commonly presenting in children and in adults between the ages of 20 and 50 years [4, 5].

The clinical manifestations of EC are often nonspecific and overlap with other GI conditions, posing diagnostic challenges. Diarrhea and abdominal pain are the most frequently reported symptoms, occurring in approximately 60%–80% of the patients. Other symptoms include nausea and vomiting (reported in ∼30% of cases) and, less frequently, rectal bleeding, which is present in 10%–20% of the affected individuals. Weight loss, when present, is typically minimal [4].

The diagnosis of EC relies on the combination of persistent GI symptoms, histopathological evidence of eosinophilic infiltration in colonic biopsies, and exclusion of alternative causes of tissue eosinophilia, including infections, inflammatory bowel disease, and drug reactions. There is currently no established consensus on the optimal treatment approach for EC. However, corticosteroids have demonstrated clinical efficacy in the majority of reported cases. For patients with refractory disease, immunomodulatory agents or biologic therapies may be considered. Surgical intervention is reserved for the management of complications such as bowel obstruction or perforation [1, 4, 6]. Here, we present a case of adult‐onset EC in a patient with a background of allergic rhinitis who presented with diarrhea and abdominal pain.

## 2. Case Presentation

A 48‐year‐old male with a background of allergic rhinitis presented with a 7‐month history of intermittent, nonbloody diarrhea, associated with lower abdominal cramping, bloating, and occasional tenesmus. He reported unintentional weight loss of 4 kg and progressive fatigue over the past month. He denied fever, night sweats, rectal bleeding, recent travel, new medications, or antibiotic use. There was no personal or family history of inflammatory bowel disease, colorectal cancer, parasitic infections, or drug allergies.

On physical examination, the patient appeared well, with stable vital signs. The examination was unremarkable except for mild conjunctival injection and tearing of the eyes. Laboratory workup showed a total white blood cell count of 8800/μL, with absolute eosinophil count of 1936/μL (22.0%). Other hematologic and biochemical parameters, including liver and renal function tests, CRP, ESR, and coagulation profile, were within normal limits. Stool microscopy and occult blood were negative. Fecal calprotectin was 9 μg/g, and abdominal ultrasound was unremarkable.

**FIGURE 1:**
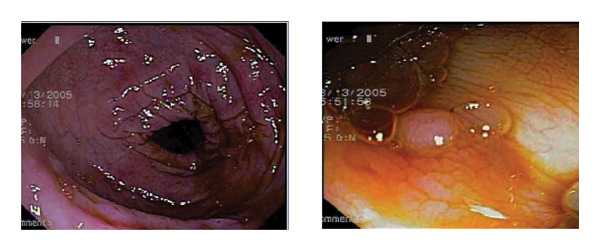
Colonoscopy image showing patchy erythematous mucosa in the rectum and colon, along with a small sessile polyp in the transverse colon.

Colonoscopy revealed patchy erythematous mucosa in the rectum and colon, along with a small sessile polyp in the transverse colon, which was excised with biopsy forceps (Figure 1). Few engorged, nonbleeding internal hemorrhoids were also noted.

**FIGURE 2:**
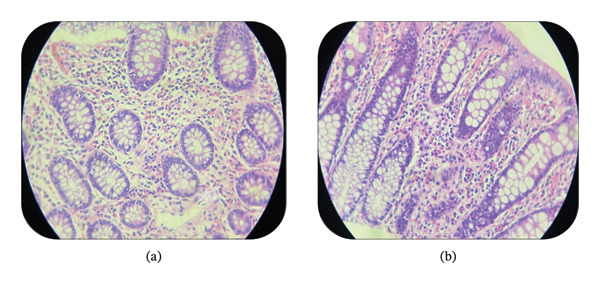
Histopathologic image showing maintained crypt architecture with dense eosinophilic infiltrates expanding the lamina propria. (a) Transverse colon. (b) Rectum.

Histopathological examination of both the transverse colon polyp and inflamed rectal mucosa showed polypoid lesion fragments with preserved crypt architecture, slight increase in lamina propria lymphoplasmacytic infiltrates, and marked eosinophilic infiltration (> 35 eos/high‐power field [HPF]) (Figure 2). No parasites or dysplasia were identified.

A diagnosis of primary EC was made based on chronic GI symptoms, histological evidence of marked eosinophilic infiltration in the colonic mucosa, peripheral eosinophilia, characteristic endoscopic findings, and exclusion of secondary causes such as infections, inflammatory bowel disease, malignancy, drug reactions, parasitic infections, and systemic eosinophilic syndromes. The patient was started on oral prednisolone 40 mg/day, tapered gradually over 8 weeks, along with antihistamines for associated allergic symptoms. At one‐month follow‐up, the patient showed significant clinical improvement with reduced stool frequency and a decrease in eosinophil count to 450/μL. By the 2‐month follow‐up, clinical remission was maintained poststeroid taper, and at six months, the patient remained in remission with a normalized eosinophil count of 150/μL, weight gain, and no steroid‐related complications.

## 3. Discussion

EC is a rare condition, with only a limited number of cases reported since 1979. It is a heterogeneous disorder characterized primarily by intense eosinophilic infiltration of the colon, which can be either segmental or diffuse in the absence of known secondary causes of eosinophilia [1, 7]. The differential diagnosis of increased eosinophil density in colonic mucosa is clinically challenging, as eosinophils are normally present in the colon, unlike the esophagus. Physiologically, eosinophil levels are highest in the ascending colon, gradually decreasing toward the rectosigmoid region, and may increase further in response to various inflammatory conditions. Since eosinophil‐rich inflammation is not unique to EC, a diagnosis of primary EC should only be made after excluding all other identifiable causes of colonic eosinophilia [2].

EGIDs are rarely reported in sub‐Saharan Africa, where parasitic infections remain the most common cause of eosinophilia [8]. A systematic review and meta‐analysis found that among adults with GI symptoms, the prevalence of noneosinophilic esophagitis EGIDs was 1.9%, with a lower prevalence observed in developing countries (1.5%) compared with developed countries (2.4%) [9]. In Ethiopia, a previously reported case of EGID involved a 25‐year‐old patient, who presented with ascites and was diagnosed with the subserosal subtype of eosinophilic gastroenteritis [10]. However, to the best of our knowledge, this is the first documented case of EC in Ethiopia.

Although its exact pathogenesis remains unclear, current evidence points to a multifactorial origin involving a dysregulated Th2 immune response. In children, EC is often IgE mediated and strongly associated with food allergies such as cow’s milk protein allergy. In contrast, adult‐onset EC is more commonly driven by a non‐IgE, CD4+ T‐cell–mediated immune mechanism [1, 5, 7]. However, a recent study suggests that Th2 inflammation may play a minimal or no role in EC pathogenesis. Instead, apoptosis and reduced epithelial cell proliferation may be responsible, indicating that EC may have a unique pathophysiology compared with other EGIDs [2].

Clinically, EC presents with a broad range of nonspecific GI symptoms. The clinical presentation varies based on the depth of eosinophilic infiltration, as described by Klein et al. Mucosal EC, the most common subtype, is associated with chronic symptoms such as abdominal pain, diarrhea, malabsorption, and protein‐losing enteropathy. Muscular or transmural EC, although less frequent, can lead to obstructive symptoms, intussusception, or even perforation due to involvement of the muscularis layer. The rarest form, serosal EC, typically manifests as eosinophilic ascites and generally follows a favorable, nonrelapsing course [1, 4, 6]. In our case, both the clinical features and histopathological findings were consistent with mucosal EC, as evidenced by chronic diarrhea, abdominal discomfort, and dense eosinophilic infiltration confined to the mucosa on biopsy.

A personal or family history of allergic disease is reported in 30%–75% of adult EC cases. The most commonly associated allergic conditions include allergic rhinitis (30%–39%), eczema (25%–30%), and asthma (15%–20%). In children, food allergies are more prominent, being identified in over 60% of the cases [4]. In our case, allergic rhinitis was the associated allergic disease.

Diagnosis is based on three essential criteria: persistent GI symptoms, histopathologic evidence of eosinophilic infiltration, and exclusion of secondary causes. However, there is no universally accepted histologic threshold for eosinophils per HPF, contributing to diagnostic variability. Suggested cutoffs include > 50 eos/HPF in the right colon, > 35 in the transverse colon, and > 25 in the left colon in symptomatic patients. The patchy distribution of eosinophilic infiltration further complicates diagnosis, necessitating multiple biopsies from different colonic regions [7, 11]. Peripheral eosinophilia may serve as a supportive marker but is neither sensitive nor specific for EC. It is inconsistently observed, reported in approximately 27%–75% of the cases, and often transient. As such, the interpretation of peripheral eosinophil counts should be contextualized within the broader clinical and histological findings [4]. In our case, the diagnosis of primary EC was made based on the patient’s persistent GI symptoms such as diarrhea and abdominal pain, histological evidence of marked mucosal eosinophilic infiltration (> 35 eosinophils/HPF), and ruling out secondary causes.

Endoscopic findings in EC are frequently subtle or absent. When present, they may include mucosal erythema, granularity, and superficial ulcerations. EC presented most commonly with erythema (12%), ulceration (4%), nodularity (5%), and mucosal friability (11%). Unique to EC, polyps (6%) can also be seen [6, 12]. Similarly, radiological signs are not pathognomonic but can aid in supporting the diagnosis. Findings such as bowel wall thickening, strictures, and the characteristic “halo sign” may reflect submucosal edema and inflammation. In case of mucosal EC, the “araneid limb‐like sign” may be seen [1, 6, 11]. In our case, colonoscopy evaluation revealed patchy erythematous mucosa and a sessile polyp in the transverse colon but abdominal ultrasound was unremarkable.

Management strategies differ based on age and severity. In children, dietary elimination of offending allergens is often effective. In adults, systemic corticosteroids remain the mainstay of therapy. Prednisone and budesonide are effective in inducing remission though relapse and steroid dependence are common, necessitating long‐term maintenance strategies. Budesonide CIR offers a targeted approach with reduced systemic toxicity. Immunosuppressants like azathioprine or 6‐mercaptopurine are considered for steroid‐refractory or steroid‐dependent cases. Surgical intervention is reserved for complications such as obstruction or perforation, more frequently observed in muscular‐type EC [1, 6].

The EC that develops in infancy carries a good prognosis. It tends to spontaneously resolve, often within days. After a few years, these young children can even tolerate the implicated foods. For young adults with EC, its natural history tends to become chronic with periods of activity and periods of apparent remission [1]. Our patient responded favorably to a course of systemic corticosteroids, achieving sustained clinical remission over 6 months.

## 4. Conclusion

EC is a rare and underrecognized cause of chronic GI symptoms. Its nonspecific presentation and lack of clear diagnostic criteria make diagnosis challenging. This case highlights the need for high clinical suspicion and inclusion of EC in the differential diagnosis of unexplained abdominal symptoms particularly in sub‐Saharan Africa. Corticosteroids are effective for treatment, but further research is needed to define diagnostic standards and long‐term management strategies.

**Nomenclature**
EC: Eosinophilic colitis
EGIDs: Eosinophilic gastrointestinal diseases
GI: Gastrointestinal
HPF: High‐power field
CRP: C‐reactive protein
ESR: Erythrocyte sedimentation rate
CIR: Controlled ileal release

## Author Contributions

Kaleb Assefa Berhane: conceptualization, investigation, methodology, data curation, project administration, writing–original draft, and writing–review and editing.

Ahmed Adem: investigation, methodology, data curation, resources, and writing–review and editing.

Abate Bane Shewaye: investigation, methodology, project administration, and writing–review and editing.

Nathan Tolemariam Jibat: visualization, supervision, writing–original draft, and writing–review and editing.

Lela Alemayehu Gebeyehu: visualization, supervision, writing–original draft, and writing–review and editing.

Fadil Nuredin Abrar: visualization, investigation, and writing–review and editing.

## Funding

No funding was received for this research.

## Disclosure

All authors reviewed and approved the final version of the manuscript.

## Ethics Statement

The study is exempt from ethical approval in our institution. All the information obtained was held confidential and used only for the intended purpose.

## Consent

Written informed consent was obtained from the patient for publication of this case report and the accompanying images.

## Conflicts of Interest

The authors declare no conflicts of interest.

## Data Availability

The data that support the findings of this study are available from the corresponding author upon reasonable request.
